# Supplementary material for: Co-Culture of Halotolerant Bacteria to Produce Poly(3-hydroxybutyrate-co-3-hydroxyvalerate) Using Sewage Wastewater Substrate
Source: Polymers (Basel). 2022 Nov 16;14(22):4963. doi: 10.3390/polym14224963 (PMC9699070; doi:10.3390/polym14224963)
Supplement: Supplementary file 1 [file polymers-14-04963-s001.zip › polymers-1975940-supplementary.pdf]

Supplementary Material: Sewage wastewater analysis

Parameter	Standard Methods	Concentration (mg/L)
[***]COD	5220-B	201.0
Magnesium	3500-B	20.0
Nitrate	4500-B	20
Nitrite	4500-B	0
Copper	3120-B	2.068
Iron	3120-B	0.52
Zinc	3120-B	5
Manganese	3120-B	0.025
Sodium	5300-B	71.0
Potassium	5300-B	12
[***]Total sugars	AOAC[*]	5300
Phosphorus	4500-B	2.0
Protein	--	0.67
Total Kjeldhal Nitrogen	4500-B	10-N
Acetic acid	HPLC	73
Propionic acid	HPLC	53
